# Structure and evolution of the magnetochrome domains: no longer alone

**DOI:** 10.3389/fmicb.2014.00117

**Published:** 2014-03-25

**Authors:** Pascal Arnoux, Marina I. Siponen, Christopher T. Lefèvre, Nicolas Ginet, David Pignol

**Affiliations:** ^1^Commissariat à l'énergie Atomique, DSV, IBEB, Lab Bioenerget CellulaireSaint-Paul-lez-Durance, France; ^2^Centre National de la Recherche Scientifique, UMR Biol Veget and Microbiol EnvironSaint-Paul-lez-Durance, France; ^3^Aix-Marseille UniversitéSaint-Paul-lez-Durance, France

**Keywords:** magnetotactic bacteria, magnetosome, cytochrome, magnetochrome, evolution, iron

## Abstract

Magnetotactic bacteria (MTB) can swim along Earth's magnetic field lines, thanks to the alignment of dedicated cytoplasmic organelles. These organelles, termed magnetosomes, are proteolipidic vesicles filled by a 35–120 nm crystal of either magnetite or greigite. The formation and alignment of magnetosomes are mediated by a group of specific genes, the *mam* genes, encoding the magnetosome-associated proteins. The whole process of magnetosome biogenesis can be divided into four sequential steps; (i) cytoplasmic membrane invagination, (ii) magnetosomes alignment, (iii) iron crystal nucleation and (iv) species-dependent mineral size and shape control. Since both magnetite and greigite are a mix of iron (III) and iron (II), iron redox state management within the magnetosome vesicle is a key issue. Recently, studies have started pointing out the importance of a MTB-specific *c*-type cytochrome domain found in several magnetosome-associated proteins (MamE, P, T, and X). This magnetochrome (MCR) domain is almost always found in tandem, and this tandem is either found alone (MamT), in combination with a PDZ domain (MamP), a domain of unknown function (MamX) or with a trypsin combined to one or two PDZ domains (MamE). By taking advantage of new genomic data available on MTB and a recent structural study of MamP, which helped define the MCR domain boundaries, we attempt to retrace the evolutionary history within and between the different MCR-containing proteins. We propose that the observed tandem repeat of MCR is the result of a convergent evolution and attempt to explain why this domain is rarely found alone.

## Introduction

Some bacteria found in aquatic environments display the singular ability to align passively along Earth's or artificial magnetic field lines while they swim. The genetically controlled biomineralization of magnetic nanocrystals makes this magnetotaxis possible. Made of iron oxide (magnetite, Fe^2+^Fe^3+^_2_O_4_) and/or iron sulfide (greigite, Fe^2+^Fe^3+^_2_S_4_), these nanomagnets are each embedded in a proteolypidic membrane, forming magnetosomes. These magnetosomes are aligned within the cytoplasm of magnetotactic bacteria (MTB), acting as a compass needle for orientation. A tentative selective advantage would be an efficient localization of the cells in vicinity of the oxic-anoxic transition zone in the water column at their preferred position in the oxygen (and perhaps redox potential) gradient. Since their first scientific description by RP Blakemore in 1975 (Blakemore, 1975), major breakthroughs in MTB isolation and cultivation, combined with advances in genome sequencing technologies have led to ever increasing amounts of information on their ecology, physiology, phylogeny, and evolution (Bazylinski et al., 2013).

Both cultured and uncultured MTB studied thus far are found within the domain Bacteria and affiliated with three phyla: (i) the *Proteobacteria* phylum with MTB belonging to the *Alpha*-, *Gamma*-, and *Deltaproteobacteria* classes, (ii) the *Nitrospirae* phylum, including several uncultured strains and, (iii) the *Planctomycetes*-*Verrucomicrobia*-*Chlamydiae* (PVC) (Lefèvre and Bazylinski, 2013). Regardless the phylogenetic affiliation, the magnetosomes biomineralized by a given species display a very narrow size range (from about 35 to 120 nm) and a given shape (e.g., cubooctahedric, elongated prismatic, bullet shaped). For any given strain, magnetosomes are aligned in chains of constant length and number along the long axis of the cell. When both greigite and magnetite are synthesized, magnetosomes loaded with either mineral are found within the same chain (Lefèvre et al., 2011). Taken together these observations suggest a tight genetic control of the molecular mechanisms governing magnetosome biogenesis. This was confirmed by every comparative genomic analyses published to date with the identification of a series of genes involved in magnetosome biomineralization, specific to and present in MTB, called *mam* (magnetosome membrane) genes. The *mam* genes are organized in clusters in the genome of MTB, in some cases defining a *bona fide* magnetosome genomic island (MAI) (Komeili, 2012). Currently, 13 of these genomic regions have been sequenced, covering all but the PVC phylum of the MTB phylogenetic tree (Grünberg et al., 2001; Matsunaga et al., 2005; Jogler et al., 2009, 2011; Nakazawa et al., 2009; Schübbe et al., 2009; Lefèvre et al., 2013a,b; Ji et al., 2013). A core gene set composed of *mamA, B, I, E, K, M, O, P* and *Q* is conserved among all MTB regardless the chemical composition of the nanocrystal, with an additional gene, *mamL*, in magnetite-producers. These genes are regrouped in *mamAB* or *mamAB-like* operons, referring to the genetic organization described in the paradigm strains *Magnetospirillum magneticum* AMB-1 and *Magnetospirillum gryphiswaldense* MSR-1 (Lefèvre et al., 2013a). These *in silico* analyses are nicely confirmed by genetic and biochemical approaches in these 2 strains where the *mamAB* operon alone is sufficient for magnetite biomineralization and magnetosomes organization (Murat et al., 2010; Ullrich and Schüler, 2010; Lohsse et al., 2011). Other than bioinformatics predictions, a very limited number of molecular mechanisms have been experimentally evidenced so far. One can cite MamK, a bacterial actin-like protein forming *in vitro* and *in vivo* filaments involved in the magnetosome chain assembly (Rioux et al., 2010; Draper et al., 2011; Sonkaria et al., 2012; Ozyamak et al., 2013), MamJ that link the magnetosome to the MamK filament (Scheffel et al., 2006; Scheffel and Schüler, 2007) and MamA that coats the outside of the magnetosome and presumably helps the localization of other magnetosome associated proteins (Zeytuni et al., 2011).

Amongst the Mam proteins, a series of predicted redox proteins exhibit a *c*-type cytochromes motif endemic in MTB and potentially play a role in the iron biocrystallization process that takes place inside the magnetosome (Siponen et al., 2012). The magnetochrome (MCR) domain contains a CXXCH motif that forms a *c*-type heme-binding site, which is only found in four proteins associated with the magnetosome (MamP, E, T, X, see Table 1 for a list of MCR containing proteins). It is usually present as a tandem repeat, rarely alone or in more repeats, and in all cases the MCR-containing proteins are predicted to be associated to the magnetosome membrane through a single membrane spanning α-helix. This original wrapping of *c*-type cytochromes inevitably suggests their participation in an electron transfer chain. Whether it concerns bioenergetics to drive iron import, manage the redox balance of the iron pool or any other molecular mechanisms requiring electron transport is still an open question. Nevertheless, recent studies on MamE, MamX, and MamP were published, hinting at potential functions for MCR domains during magnetosome biogenesis.

**Table 1:** **list of MCR containing proteins**.

**Bacteria**	**MamE**	**MamP**	**MamT**	**MamX**	**Other**
**AMB-1**	3^(*)^	1	1	1	
**MSR-1**	1	1	1	1	
**MS-1**	2	1	1	1	
**MC-1**	1	1	1	1	
**MV-1**	1	1	1	1	
**QH-2**	1	1	1	1	
**SS-5**	2^(§)^	1	1	-	
**RS-1^$^**	1^(†)^	-	-	-	MamP^*(‡)^
**BW-1**	1^(†)^	-	-	-	MamP^*(‡)^
**M. bavaricum**	1^(¶)^	1	^(#)^	^(#)^	

* *Three MamE paralogs with small variations: The “classical” MamE (amb0963) with two MCR domains, MamE-Like (amb0410) and LimE or Like-MamE (amb1002) with four MCR domains*.

† *Different from the classical MamE with the PDZ domain replaced by a TauE domain (Trypsin-MCR1-MCR2-TauE)*.

‡ MamP^*^ is different from MamP or MamT but contains two putative MCR domains with the following architecture: MCR1-MCR2-PDZ-NitroFeMoCo.

# *Homolog absent but the entire genome has not been sequenced yet*.

§ *Two paralogs of MamE with one (MamE) containing four MCR domains (MCRA1-MCRA2-Trypsin-MCR1-MCR2-PDZ) and the other (MamE') containing only one MCR domain between the Trypsin and the PDZ domains (Trypsin-MCR0-PDZ)*.

¶ *Contains only one MCR domain (Trypsin-MCR0-PDZ)*.

In a recent study focused on the biochemistry of MamP and its structural characterization, it was found that MamP displays ferroxidase activity (Siponen et al., 2013). Because of the presence of ferric reductases in MTB (Zhang et al., 2013), as well as the presence of ferrous diffusion facilitators encoded in the MAI (Uebe et al., 2011), Fe(II) is likely the most readily available form of iron for crystal growth. Since both magnetite and greigite are a mix of iron(III) and iron(II), this implies the presence of Fe(II) oxidation occurring in the magnetosome. MCR-containing proteins such as MamP would be involved in the control of the Fe(II) and Fe(III) ratio required for magnetite biomineralization (Siponen et al., 2013). This function is supported by *in vitro* mineralization experiments. Thus, MamP is able to induce magnetite mineralization in the sole presence of Fe(II), whereas chemical synthesis requires mixing iron(II) and iron(III) in appropriate proportion (Baumgartner et al., 2013a). MamP ferroxidase activity is then sufficient to produce the iron(III) required for magnetite growth. Siponen et al. observed that the initial formation of the mineral phase is ferrihydrite (an iron(III) oxide), magnetite appearing later in the assay. This suggests that MamP could be involved in ferrihydrite production, an intermediate of magnetite detected *in vivo* (Baumgartner et al., 2013b; Fdez-Gubieda et al., 2013). Further work using different species is required to firmly establish the role of MamP *in vivo*, and to determine its electron transfer partner(s).

The redundancy of MCR domains across different proteins of the magnetosome membrane can make their functional characterization somewhat difficult. This is particularly true for the laboratory strain *Magnetospirillum magneticum* AMB-1, where multiple paralogs of Mam proteins exist. As a consequence, deletion of the MCR domains in one protein might be compensated by the presence of another MCR-containing paralog. This is well illustrated in the study by Quinlan et al. recently published on MamE in this strain (Quinlan et al., 2011). This protein is predicted as a protease belonging to the HtrA/DegP proteases family and is found in every genome of magnetite-producing MTB known to date. Canonical HtrA/DegP proteases possess a trypsin domain followed by two PDZ domains. A variation of this domain organization is found in MamE with the insertion of tandem MCR domains between trypsin and PDZ domains. In *M. magneticum* AMB-1, the deletion of *limE*, a paralog of *mamE*, has no phenotype, but when *mamE* is also deleted, there is a complete loss of magnetite biomineralization, although empty magnetosomes still form chains within the cytoplasm. Trans-complementation of this double mutant with a full *mamE* restores the wild-type phenotype whereas *mamE* mutants impaired in the fixation of the two *c*-type hemes only partially complemented the mutant (Quinlan et al., 2011). Complementation with a *mamE* variant impaired in its protease activity did not restore the wild-type phenotype. These observations suggest that the MCR tandem in MamE possesses a limited role in magnetite formation and that the protease function of MamE has a dominant function in crystal nucleation initiation. A search for MCR in this strain however reveals that, beside the MamP, T, X, and MamE, two paralogs of MamE are located elsewhere, one in the magnetosome island (Amb1002; named LimE for Like-MamE Quinlan et al., 2011; 63% identity with MamE), and another one present in a genomic islet that contains homologous *mam* genes distinct from the magnetosome island (Amb0410, named MamE-like Rioux et al., 2010; 53% identity with MamE). It is therefore possible that the functions of the MCR domains of MamE are maintained by the other MamE-like proteins, especially if one considers that one of these proteins (Amb0410) is an out-group in the MCR-containing family of proteins, as it possesses four MCR domains instead of the classical tandem usually found (see below). Further work is needed to clarify the functional roles of the MCR domains of MamE.

The situation is somehow clearer in *M. gryphiswaldense* strain MSR-1 in which only MamE, P, T, and X are predicted to possess two MCR domains, with no paralogs inside or outside the magnetosome island. Recently, the role of MamX was investigated in this species (Raschdorf et al., 2013). MamX is associated to the magnetosome membrane and contains a pair of MCR domains. The authors observed the presence of rare wild-type like magnetite crystals flanked by poorly crystalline particles in a Δ*mamX* strain. These “flake-like” particles were identified as hematite (Raschdorf et al., 2013). Both magnetite and hematite particles evolved concomitantly, suggesting that hematite is not an intermediate in magnetite formation and rather that the fate of these individual particles was determined at an early stage. Trans-complementation of the Δ*mamX* strain yielded a WT phenotype whereas complementation with a variant of MamX devoid of the MCR domains did not restore the WT phenotype. Together with *mamY* and *mamZ*, *mamX* belongs to the *mamXY* operon, which is a signature of magnetotactic *Alphaproteobacteria*. Its neighbor MamZ contains a predicted ferric reductase domain fused to a transporter belonging to the major facilitator superfamily (MFS). The phenotypes of the *mamX*, *mamZ* and *mamH* mutants led the authors to propose a functional MamXZH interaction that would form an iron oxidoreductase and transport complex through the magnetosome membrane. The understanding of this system and the role of the MCR-containing protein MamX need further study. For example, electron transfer partners and directionality of electron flow remain unknown for MamX.

Altogether the bioinformatics and experimental data available on MCR-containing Mam proteins suggest their involvement in iron redox chemistry to ensure the proper mineralization of magnetosomes. By taking advantage of new genomic data available and recent structural data on MCR domain, we attempt to retrace the evolutionary history of this domain within and between the different MCR-containing proteins. We also hypothesize on the reasons why this domain is rarely found alone but rather in tandem repeats.

## Results

### Structure of MCR

**Figure 1:**
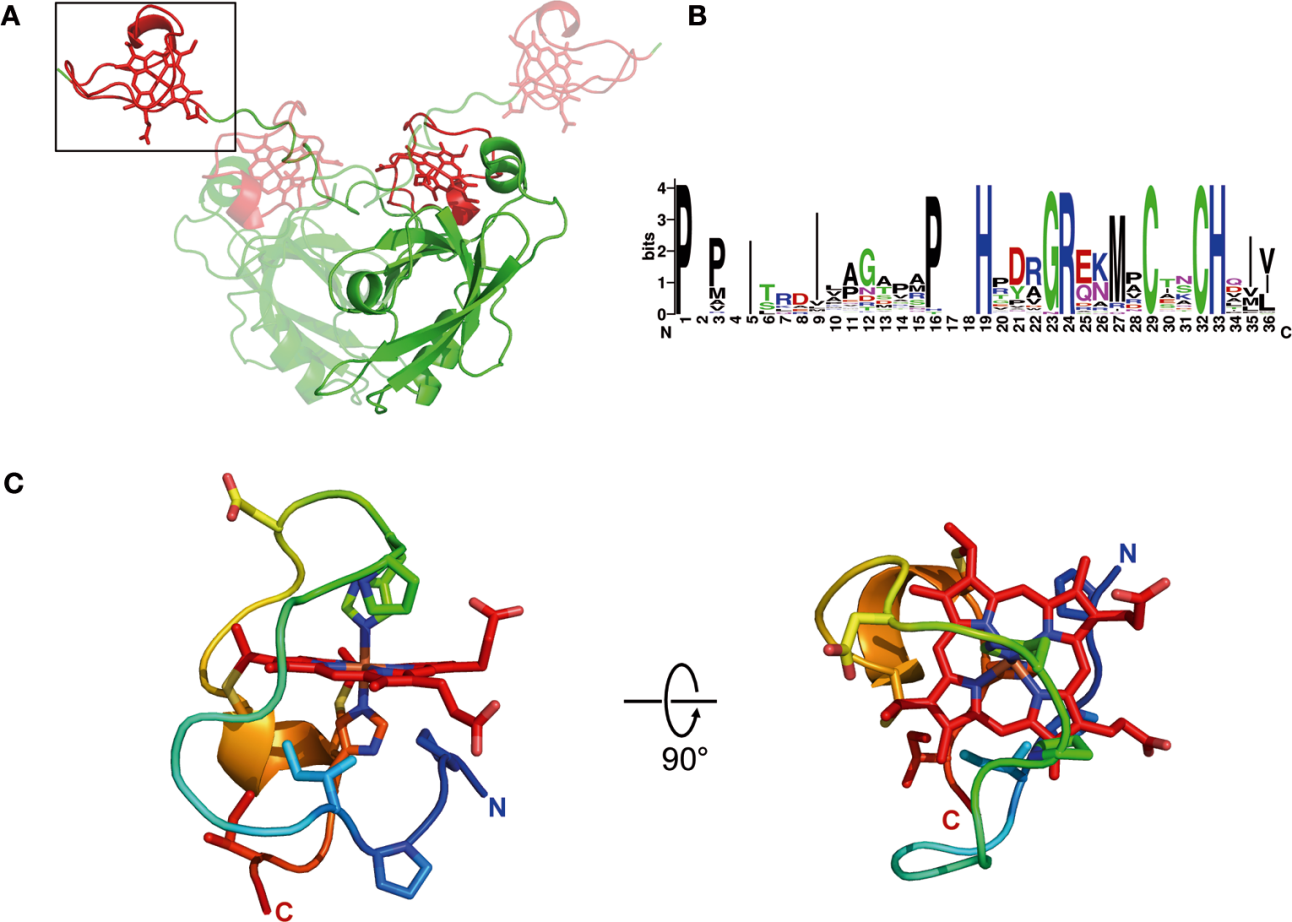
**Structure of MCR**. **(A)** Overall structure of the MamP dimer with both monomers colored according to their domain organization (PDZ in green and magnetochrome domains in red), with one monomer rendered in transparency. **(B)** Weblogo (http://weblogo.berkeley.edu/) representation of a typical magnetochrome domain. **(C)** Structure of a magnetochrome domain colored from blue (N-Terminus) to red (C-Terminus) and with a few residues conserved in the Weblogo representation shown in stick.

Newly available information on the structure of the MamP protein from the ovoid magnetotactic bacterium MO-1 has laid the groundwork for understanding the structural basis of MCR function (Siponen et al., 2013). Prior to this X-ray structure determination work, the *c*-type cytochrome domains of MamP were already proposed to define a novel domain that is only found in MTB (Siponen et al., 2012). The primary structure suggested that in MamP, a PDZ was followed by two CX_2_CH heme attachment motifs, defining two magnetochrome domains (MCR1 and MCR2). The overall fold of MamP in the crystal revealed a dimer with both monomers mainly stabilized by numerous contacts between their PDZ domains. The first magnetochrome domain (MCR1) is in contact with its own PDZ domain, while the second (MCR2) is projected above the PDZ domain of the other monomer. This structural study allowed the first fold description of a MCR domain, substantiating its uniqueness at the structural level. Indeed, a structural homology search with DaliLite v.3 returns no significant hits, demonstrating the specificity and the uniqueness of these domains (Holm and Park, 2000). Examining the MCR domains in the structure reveals that each MCR clearly defines a single domain, confirming that the MCR is a mono heme *c*-type cytochrome domain and not a diheme as it may have been inferred from its seemingly repeated structure (Figure 1A).

Based on bioinformatic analysis the minimal unit defining the MCR domain can be described as [P/T/H]HX_5−9_CX_2_CH. A more in-depth structural analysis suggests that the entire MCR domain is composed of 20 amino acids in MamP (see Materials and Methods section). A detailed examination of the structure identified two hydrophobic residues, which delineate the N-terminal and C-terminal regions of the MCR domain. In the fold, these two residues interact hydrophobically to close off the domain. Based on these observations, we proposed a more accurate delineation of a typical MCR domain: ψ 1X_5−9_PHX_5−9_CX_2_CHψ2 (Figure 1B). The MCR domain starts with a hydrophobic residue (ψ1) directly contacting the heme moiety. This is followed by a PH motif providing the 6th heme ligand and located five residues upstream (in MamP) of the CX_2_CH motif anchoring the heme to the polypeptide. Finally, the terminal hydrophobic residue (ψ2) closes the MCR fold by interacting with the ψ1 residue. Being composed of 19–28 residues, it represents the smallest mono-heme cytochrome known to date (the mono-heme cytochrome *c*-553 from *Bacillus pasteurii* contains 71 residues surrounding the heme moiety). Overall, this results in a highly solvent-exposed heme moiety, with all four solvent edges exposed (Figure 1C).

As previously mentioned, with the exception of MamT, MCR domains are often found in conjunction with other types of domains. In MamP, the MCR domains are C-terminal to a PDZ protein-protein interaction domain. The fold observed in the crystal for the entire protein is dimeric showing that the MCRs provide a redox gateway above the crucible formed by the interaction of both PDZ domains (Figure 1A). While structural information is still unavailable for the MCR domains of MamX, MamE, and MamT, the fold of the MCR in itself is likely to be very similar. However, it is noteworthy to mention that in the case of MamE, the two MCR domains may adopt a different spatial orientation as that in MamP since there is often a consequent amino acid insertion between both MCRs (30–60 amino acids depending on species). Furthermore, in the case of MamE, the MCR domains are flanked N-terminally by a protease domain and C-terminally by two PDZ domains making it difficult to predict any structural information based on the MamP structure. The MCR domains of MamX could hypothetically form a redox gateway above its domain of unknown function, as seen in MamP, but no substantial evidences exist to support this scenario. Only new structural data on these proteins will allow understanding of the overall organization of MCR within their corresponding proteins.

## Evolution of MCR domains

Among the questions about MCR evolution, we are concerned about their occurrence. For example why MCR domains are almost always found in tandem and so rarely alone or repeated more than twice (Table 1)? And for each tandem, are the two repeated MCR domains similar or not? Did they evolve from a single ancestral tandem of MCR domains or rather evolved from independent duplication events? Such intriguing questions can be approached through the evolutionary history of these MCR domains. Because the MCR domain is “endemic” in MTB, tracing back their evolutionary history should be simplified and, as it is found in the core genes set common to all MTB, we are expecting a reasonable diversity in our sample population. The structural studies on MamP described above allow a clear delineation of the domain's boundaries, which should also simplify the constitution of our sample population.

**Figure 2:**
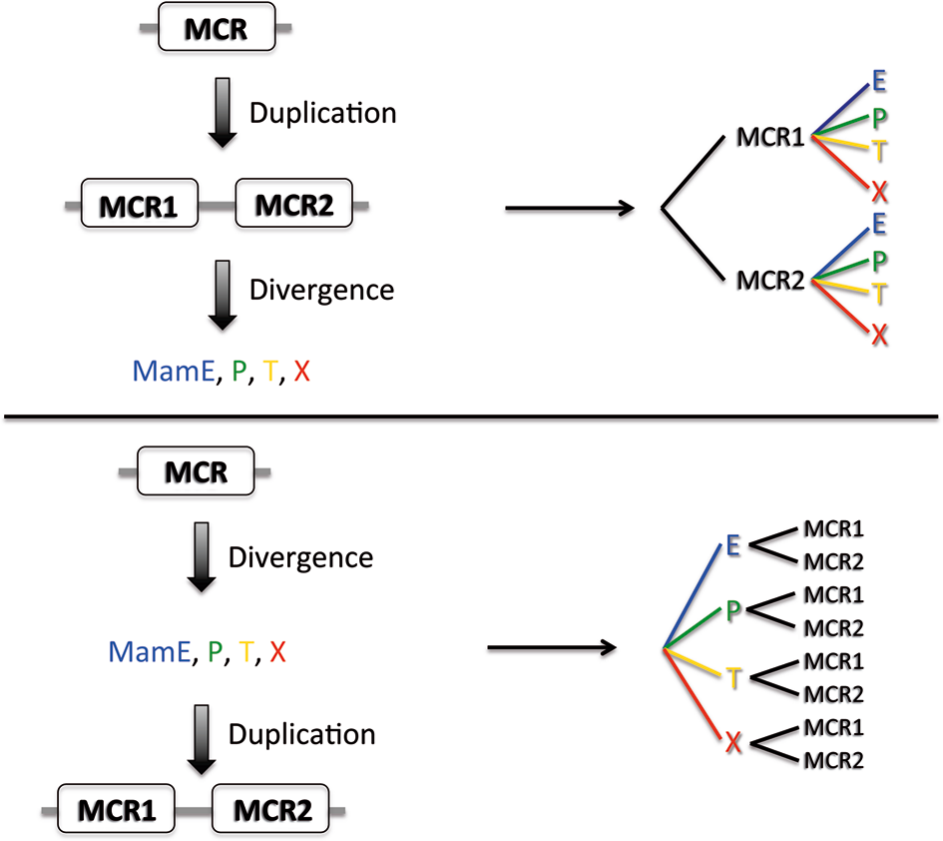
**Two models of MCR evolution**. Two putative models for MCR evolution, one where the MCR domain initially duplicated and then diverged (top) and one where the MCR domain diverged before duplication.

The evolution of a duplicated domain can be considered in two simple evolutionary models where internal duplication of the original domain takes place either before (Figure 2, Model #1) or after (Figure 2, Model #2) functional and sequence divergence of the entire protein. In the first case, MCR1 and MCR2 domains would appear as two separate branches in a phylogenetic tree whatever the protein considered (MamE, P, T, X), whereas in the second case the separation would initially occur between the proteins containing the MCR domains forming separate branches for (MamE, P, T, X). At first sight, the first model seems the simplest to explain the functional diversity observed in MCR-containing proteins. Indeed, an initial (and presumably rare) event of internal domain duplication would have taken place, followed by a functional divergence of the proteins. The second model is probably less intuitive as it depicts a single MCR divergence before the duplication events; however this model does not explain why the domain is rarely found alone but almost always in duplicate, unless we think about convergent evolution. An alternative model explaining why the MCR1 and MCR2 domains share more sequence identity within a family would be that there is a evolutionary constraint on the MCR1 and MCR2 that must be kept similar to each other for the dimer to be functional.

**Figure 3:**
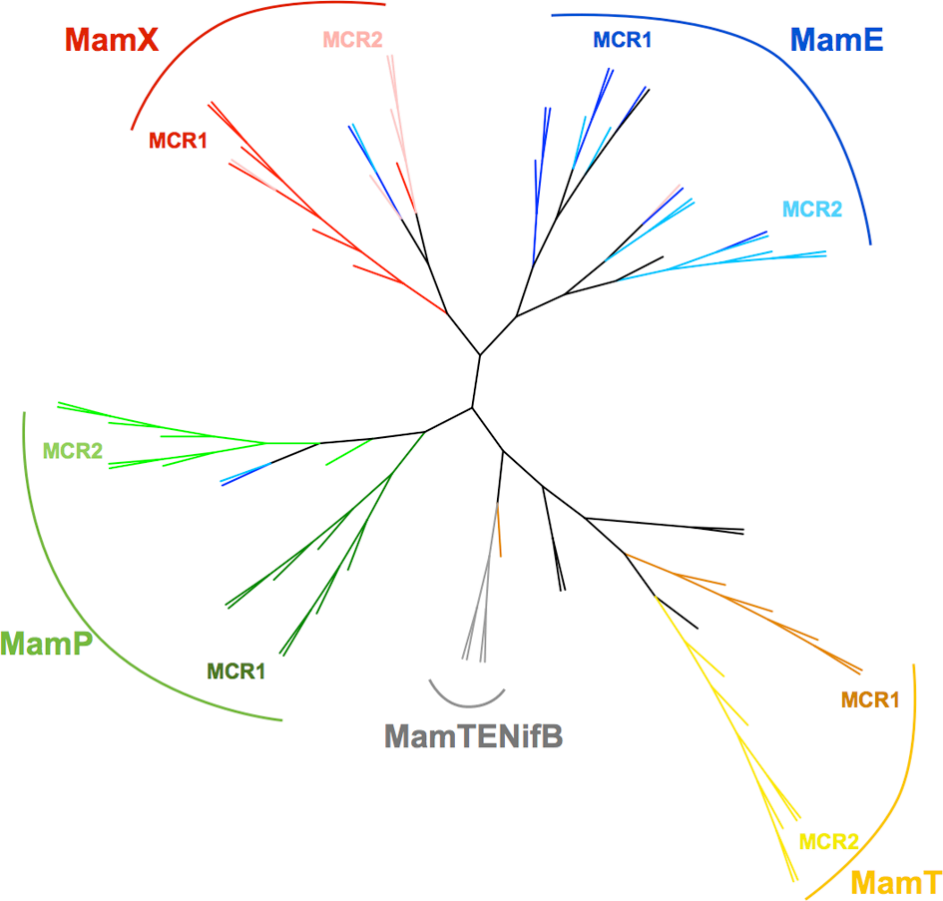
**Phylogenetic tree of MCR domains**. The tree with the highest log likelihood (-1151.8298) is shown. The tree is drawn to scale, with branch lengths measured in the number of substitutions per site. The analysis involved 88 amino acid sequences. All positions containing gaps and missing data were eliminated. There were a total of 19 positions in the final dataset. Evolutionary analyses were conducted in MEGA5 (Tamura et al., 2011). Tree was edited and drawn with the interactive tree editor iTOL (Letunic and Bork, 2011). Color of the branches is according to the MCR domain position and the Mam protein it belongs to. For MCR 1 and MCR2 of MamE branches are respectively blue and light blue, MamP, green and light green; MamT, orange and yellow; MamX, red and salmon; MamTENifB, gray. Out-groups are left black.

To generate our dataset, we gathered sequences of MCR containing proteins MamE, P, T, and X from 10 species (see Materials and Methods) and separated them into MCR1 and MCR2 as described above. A protein sequences alignment was computed with all the individual MCR using the Muscle algorithm (Edgar, 2004) and a phylogenetic trees built with MEGA5 software (Tamura et al., 2011); the MCR alignment as well as the individual protein sequence alignment are provided as Supplementary Figure 1. The resulting tree presented in Figure 3 displays several branches with clear boundaries, which is already surprising considering the short size of the MCR domains (19 amino acids) and the low bootstrap values when generated (data not shown). Much to our surprise, we found that the MCR domains do not clusterize according to their position in the amino acid sequence (MCR1 or MCR2) as expected for model #1 but rather form a cluster with the Mam protein they belong to, as predicted in model #2. For instance in the case of MamE and MamT, the MCR domains, regardless of their numbering, form distinct leaves for each protein. Then within each leaf we observe distinct branches leading to the MCR1 and MCR2. This topology is clearly reminiscent to model #2 where divergence of the original MCR domain occurred before the internal duplication.

## Discussion

A rather simple evolutionary scheme can be proposed for the MCR-containing Mam proteins where the basic scheme is provided by model #2: an initial sequence divergence event followed by domain duplication. It is interesting to note that even based on short MCR domain sequences, one can relatively easily infer the nature of the protein to which it belongs (MamE, P, T or X).

Whether it is an ancient or more recent evolution, whether or not it is part of the minimal gene set required for magnetosome biosynthesis, the major trend for the magnetochrome domain evolution is a tandem duplication after a sequence divergence. It seems that when a new protein with a tandem MCR domain is selected by evolution, it always evolves from a lone MCR domain and not from an existing tandem repeat. For example, it is known that MamX is only present in MTB from the *Alphaproteobacteria*, suggesting that it evolved relatively recently. However, its evolutionary history based on the MCR domain only suggests that it did not emerge from the tandem of another MCR-containing protein like previously existing MamP or MamE. What we may be witnessing here is an example of convergent evolution where the tandem repeat is linked to the functional role of the protein. Indeed, this domain is almost always found in tandem and there are only rare examples where it is found either, alone (MamE of *Candidatus Magnetobacterium bavaricum* and MamE' of SS-5) or in triplicate (MamE of strain MC-1). It is tempting to link this pattern to iron and magnetite (or greigite) chemistry. Both magnetite and greigite are a mix of one iron(II) and two iron(III) equivalents. The possibility to abstract or give two electrons by a pair of magnetochrome domains suggests its involvement directly in magnetite or greigite crystal production, not just the iron chemistry that requires a single electron. Such use of two monoheme cytochromes was also suggested to evolve in order to adapt to the storage of the two electron generated from sulfite oxidation (Robin et al., 2013). Although this hypothesis of two MCR domains adaptation to a two-electron transfer reaction is tempting, it will be difficult to test and further work is definitely needed in order to better understand the complex connections between MTB and MCR.

A hypothesis concerning the evolution of magnetotactic bacteria suggests they all evolved from a common ancestor 2.5–3.0 billion years ago when levels of atmospheric oxygen were low and anaerobic to microaerobic environments dominated. At this time, magnetite/greigite crystals likely did not serve a role in magnetotaxis but rather in scavenging reactive oxygen species and later, when atmospheric oxygen levels increased, served to aid MTB in navigation (Lefèvre et al., 2013a). This ancestor probably had only the *mamAB* operon (including *mamE* and *mamP*) and the magnetite/greigite crystals have diversified since then. Because magnetochrome-containing proteins are the only redox proteins associated to the magnetosome this raises interesting possibilities possibly linking magnetite/greigite crystal shape to the evolution of these magnetochrome-containing proteins.

The three dimensional structure of MamP showed that the first magnetochrome domain contributed to the formation of a crucible in which iron could be stabilized (Siponen et al., 2013). This structural study enabled a better analysis of the evolutionary history of MCR domains by defining the boundaries of this domain. The structure of other MCR-containing proteins such as MamE or MamX will allow to better define the role of the magnetochrome domain in the context of magnetotaxis. Furthermore, these studies will also allow more robust structure-based sequence alignments. Finally, interesting questions that need to be answered in the future relate to the interaction between these MCR containing proteins and the identification of their electron-transfer partners.

## Methods

The complete genomes or contigs discussed in this paper include: *Magnetospirillum magneticum* AMB-1 (GenBank: NC_007626.1), *Magnetospirillum gryphiswaldense* MSR-1 (GenBank: CU459003.1), *Magnetospirillum magnetotacticum* MS-1 (JGI project 402922), *Magnetococcus*sp. MC-1 (GenBank: CP000471), *Magnetovibrio blakemorei* MV-1 (GenBank: FP102531), *Magnetospira* sp. QH-2 (EMBL: FO538765), strain SS-5 (MamP: JX628772, MamE: JX628767), *Candidatus Magnetobacterium bavaricum* (Jogler et al., 2011), *Desulfovibrio magneticus* RS-1 (NC_012795–NC_012797), *Candidatus* Desulfamplus magnetomortis BW-1 (GenBank: JN830627–JN830646 and JN845570–JN845575).

From a structure based sequence alignment we devised a protein pattern that harvests almost all the magnetochrome domains without many false positive. This pattern is the following:

$$\begin{array}{l} \left[\text{P}]-\text{x}(\text{0},\text{6})-[\text{IVMLQA}]-\text{x}(\text{6})-[\text{PTH}]-\text{X}(\text{0},\text{2})-[\text{H}\right]\\ -\text{x}(1,3)-[\text{GN}]-\text{x}(1,5)-\text{C}-\text{x}-\text{x}-\text{C}-\text{H}-\text{x}-[\text{IVMLFY}] \end{array}$$

and much of the false positive belong to a single protein subunit, NrfB from a formate dependent Cytochrome *c* nitrite reductase.

Initial multiple alignment were generated using the MUSCLE program (Edgar, 2004), applying the default settings. Alignment were visualizes using Jalview package. Evolutionary trees were obtained using the MEGA5 package (Tamura et al., 2011) and by using the Maximum Likelihood method based on the JTT matrix-based model (Jones et al., 1992). Initial tree(s) for the heuristic search were obtained automatically by applying Neighbor-Join and BioNJ algorithms to a matrix of pairwise distances estimated using a JTT model, and then selecting the topology with superior log likelihood value.

### Conflict of interest statement

The authors declare that the research was conducted in the absence of any commercial or financial relationships that could be construed as a potential conflict of interest.

## Supplementary material

Supplementary Figure 1 **Sequence alignment of MCR domains used in this analysis**. Sequence alignment is colored as defined by clustalx in Jalview 2 (Waterhouse et al., 2009).
