# Supplementary figures and images for: Structure and evolution of the magnetochrome domains: no longer alone
Source: Front Microbiol. 2014 Mar 25;5:117. doi: 10.3389/fmicb.2014.00117 (PMC3971196; doi:10.3389/fmicb.2014.00117)

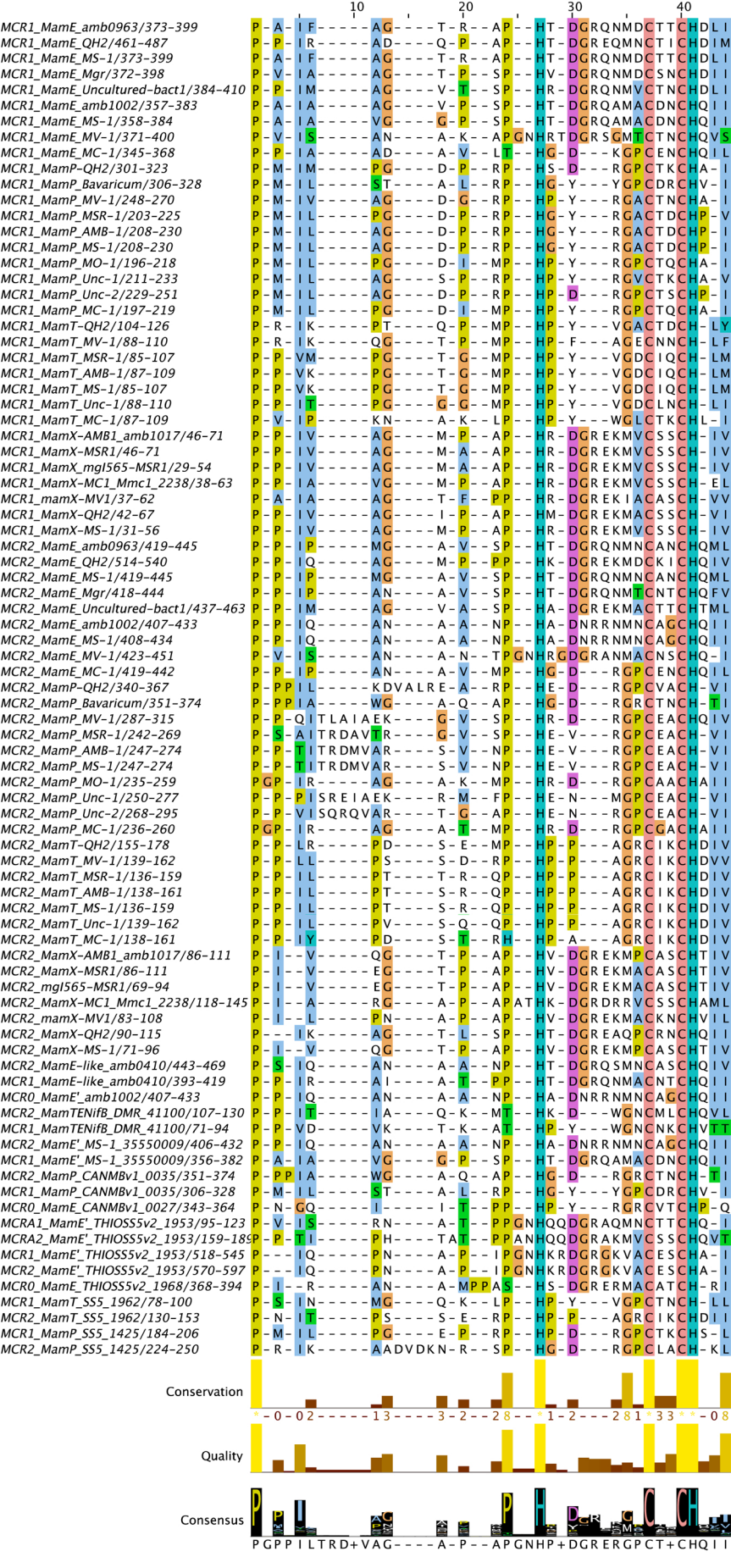

Supplement: Supplementary Figure 1 — Sequence alignment of MCR domains used in this analysis. Sequence alignment is colored as defined by clustalx in Jalview 2 (Waterhouse et al., 2009). [file Presentation1.PDF]
